# Heat generation in spatially confined solids through electronic light scattering

**DOI:** 10.1515/nanoph-2025-0118

**Published:** 2025-06-19

**Authors:** Sergey S. Kharintsev, Elina I. Battalova

**Affiliations:** Department of Optics and Nanophotonics, Institute of Physics, 64922 Kazan Federal University, Kremlevskaya Str., 16a, Kazan, 420008, Russia

**Keywords:** electronic light scattering, electron-photon interaction, optical heating, indirect transitions, nonlocal photonics, photon momentum

## Abstract

This study focuses on the optical heating of spatially dispersive solids due to electronic light scattering (ELS), a phenomenon driven by indirect optical transitions. In this process, a light-illuminated spatial heterogeneity generates an optical near-field photon with expanded momentum and thereby electron-photon momentum matching can be fulfilled. It results in indirect optical transitions which contribute to broadband inelastic emission, a physical process known as electronic light scattering or Compton scattering of visible photons. This is followed by thermalization of the electron system, making the solids to heat up and eventually melt. We experimentally demonstrate this effect by optical melting a spatially confined semiconductor (Si) and metal (Au) under moderate continuous-wave laser illumination with the intensity of only a few MW/cm^2^. We claim that ELS represents the dominant physical mechanism governing the interaction of light with spatially dispersive media, underpinning a broad range of thermo-optical phenomena and applications.

## 1 Introduction

Everyday intuition suggests that optical heating of materials is directly associated with light absorption. This physical process plays a paramount role in most areas of photonics, optical spectroscopy and thermo-optical applications [1], [2], [3], [4], [5].

**Figure 1:**
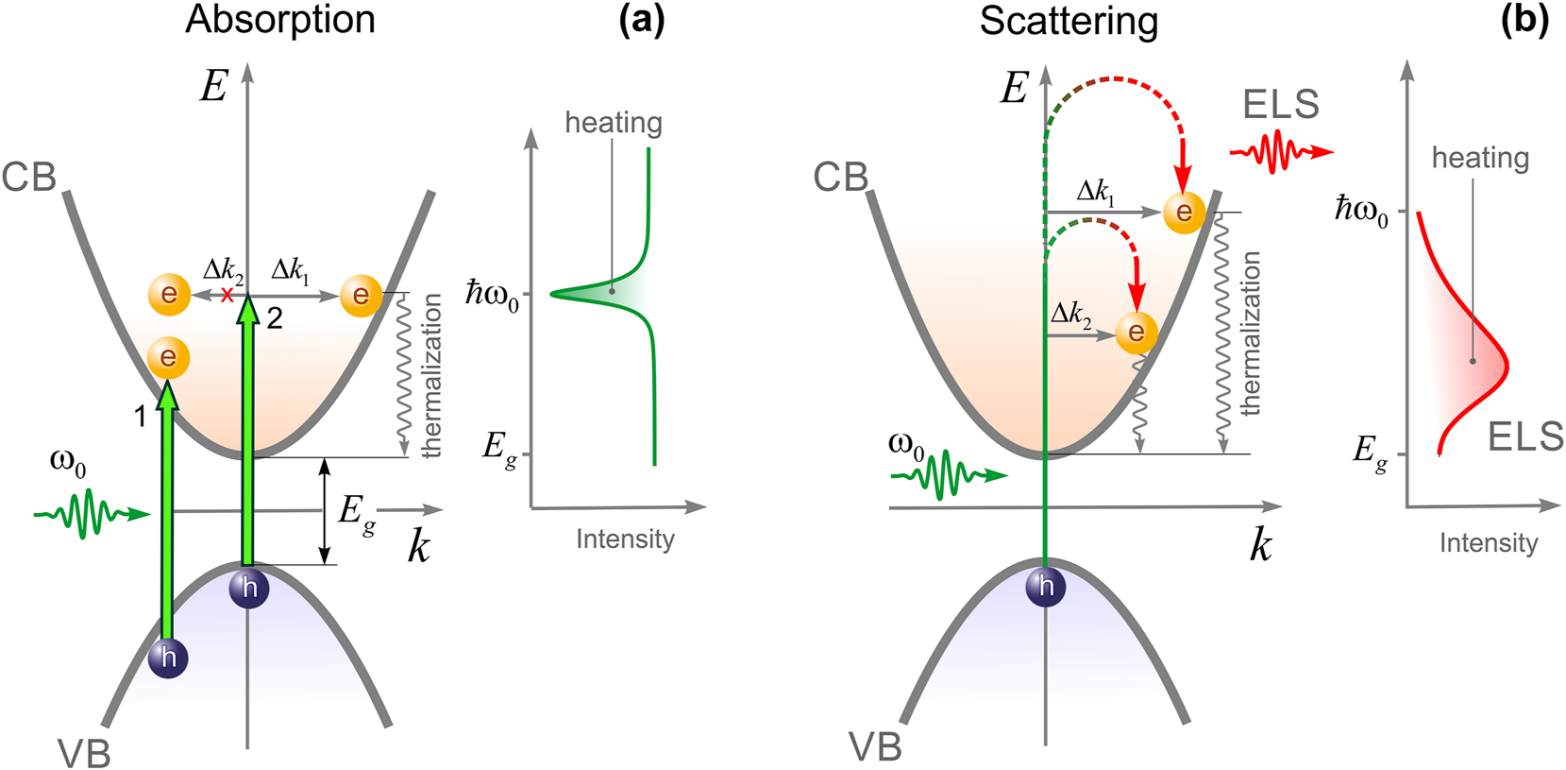
Schematic illustration of direct/indirect absorption (a) and scattering (b) in a direct bandgap semiconductor. The inset on each plot shows a spectrum of transmission (a) and reflection (b).

In a homogeneous medium with the complex permittivity $\varepsilon \left(\omega ,k\right)=\varepsilon^{\prime }\left(\omega ,k\right)+i\varepsilon^{\prime \prime }\left(\omega ,k\right)$ (*ɛ*′ and *ɛ*″ are the real and imaginary parts of permittivity, *ω* and *k* are a frequency and wavenumber of an incident electromagnetic wave), the optical heating is governed by the absorbed power in volume *V*:

(1) $$P_{\mathrm{abs}}=\underset{V}{\int }\omega \varepsilon_{0}\varepsilon^{\prime \prime }\left(\omega ,k\right){\left|E\left(\omega ,k\right)\right|}^{2}dV,$$

where ${\left|E\left(\omega ,k\right)\right|}^{2}$ is the electric field intensity, *ɛ*_0_ is the permittivity in vacuum. Since *ɛ*′′ = 2*nκ* [6], there are two contributions to material optical losses: 1) resonant absorption and 2) inelastic free-electron Drude scattering, which are determined by the extinction coefficient *κ* and the real refractive index *n*, respectively [7]. Light is absorbed by the medium through direct optical transitions (*A*_*d*_) between the electronic states near the bandgap, labelled as ‘1’ in Figure 1a. The attenuation of the intensity of the EM wave propagating through a homogeneous medium over a distance *x* exceeding its wavelength *λ*, obeys the Beer–Lambert law $I\left(x\right)=I_{0}\exp\left[-\alpha x\right]$ , where *I*_0_ is the incoming intensity inside the medium, *α* is the absorption coefficient defined as *α* = 2*ωκ*/*c* (*c* is the speed of light in vacuum) [6]. It is important to emphasize that light absorption in opaque homogeneous media is the dominant physical process compared to light scattering since their intensities are determined by the first and second orders of perturbation theory, respectively [8].

In heterogeneous media consisting of subwavelength monophase structures standard perturbation theory is no longer applicable, since it requires taking into account spatial dispersion. This effect occurs due to charge polarization near spatial inhomogeneities that generate optical near-field photons (see details in Section I in the Supporting Information (SI)) [9]. According to Heisenberg’s principle, the inherent spatial confinement of near-field photons expands the uncertainty of their momenta and, therefore, electron-photon momentum matching can be fulfilled. As a result, the real refractive index steeply climbs at the sub-wavelength scale and, in the limit of *r* ≫*λ*, asymptotically tends to its bulk value encountered in ray optics (Section II, the SI). For example, the increased real refractive index beyond the fundamental limit [10] has recently been observed in self-assembled gold nanoparticles (*n* = 10.02) [4], in perovskites during a phase transition (*n* = 26) [11], and in a graphene monolayer near a quantum well (*n* > 100) [12]. By the Kramers–Kronig relation, the extinction coefficient *κ* should tend to zero and, thereby, the heterogeneous medium becomes “transparent”. However, these conclusions contradict previously obtained results on the anomalous increase in light absorption (two orders of magnitude!) by a Si layer only 2 nm thick [13]. In this work, the authors calculated the total absorption from the standard relationship: *A*_*t*_ = 1 − *R* − *T* (where *R* and *T* is reflection and transmission). The underlying idea to explain this effect has been the indirect absorption, *A*_*in*_, labeled as ‘2’ in Figure 1a, and, thereby, *A*_*t*_ = *A*_*d*_ + *A*_*in*_ [14], [15]. Although indirect transitions can contribute to extra absorption [16], their efficiency is unlikely to be high because of energy band *k*-dispersion.

Due to the increased real refractive index, the total losses *A*_*t*_ in heterogeneous media include not only direct/indirect absorption, and also direct/indirect scattering, namely: *A*_*t*_ = *A*_*d*_ + *A*_*in*_ + *S*_*d*_ + *S*_*in*_ (where *S*_*d*_ and**** *S*_*in*_ are direct and indirect scattering). Obviously, that all these processes are convoluted and their decomposition into separate contributions is a challenging task. Absorption-free indirect light scattering *S*_*in*_ was, for the first time to our knowledge, explored in disordered perovskites under cw sub-band pump [3]. There is demonstrated that dc-pulsed-induced thermal damage of a perovskite crystal forms a glass/crystal junction at which both increased broadband emission and amplified electric current are observed. These phenomena were explained by generating a near-field photon with expanded momentum near defects [3].

The inelastic broadband emission is close to electronic Raman scattering in which initial and final electronic states are different, and optical transitions can be indirect due to electron-photon-momentum matching [5]. Though a theory of electronic Raman scattering was developed by M.V. Klein [17] back in 1983, a first experimental observation of inelastic broadband emission from a rough silver thin film was done by A.G. Mal’shukov et al. in 1989 [18]. The origin of the parasitic broadband background observed in surface-enhanced Raman scattering (SERS) spectra was attributed to high-energy electronic Raman scattering by J. Baumberg et al. in 2010 [19]. Later, in 2017, the concept of low-energy electronic Raman scattering was successfully exploited by L. Brus et al. [20] for explaining a central Raman peak originating from a CsPbBr_3_ perovskite at room temperature. As of today, this phenomenon is widely used for structural analysis of heterogeneous and/or disordered solids [5], [21] and has potential for further photo-electrical and thermo-optical applications [3].

This work focuses on the optical heating of spatially confined solids due to electronic light scattering (ELS) [5]. Herein, we claim that indirect scattering from heterogeneous media is the dominant mechanism in light–matter interactions. This statement is directly evidenced by optical melting spatially-confined silicon and gold using a tip-enhanced Raman spectroscopy (TERS) setup. The ELS is specifically the physical mechanism that unravels a number of unusual optical and spectroscopic phenomena in heterogeneous media that are still poorly understood. These include the optical melting of spatially confined solids under continuous-wave (cw) sub-band pumping [13] and the nonlinear increase in blue-shifted Raman intensity upon optical heating [22], [23].

### 1.1 Theoretical backgrounds

Optical heating of an opaque homogeneous medium is mostly contributed by direct absorption of light when resonant (band-to-band) pumping, labelled as ‘1’ in Figure 1a. Nobusada et al. demonstrated the enhanced absorption due to indirect optical transitions in a spatially-confined structure [14]. For the sake of simplicity, let us consider a heterogeneous medium composed of only two sub-wavelength structures with the sizes of *r*_1_ and *r*_2_ (*r*_1_ < *r*_2_ ≪ *λ*). Such a system absorbs and scatters light poorly since the cross-section of absorption, *C*_*a*_, and scattering, *C*_*s*_, scales with the size as *C*_*a*_ ∼ *r* ^3^ and *C*_*s*_ ∼ *r* ^6^ [24], rapidly disappearing as *r* → 0. On the other hand, these optical inhomogeneities generate near-field photons, which allow changing the momentum of an electron by Δ*k*_1_ and Δ*k*_2_ (Δ*k*_1_ > Δ*k*_2_) during its transition from the valence band (VB) to the conduction band (CB) (Figure 1a). According to Fermi’s golden rule, the strength of optical transitions is driven by not only the interaction Hamiltonian but also the electronic density of states that is maximum at the energy band edge [14]. This means that only the indirect transition is realized during which the momentum of the near-field photon, Δ*k*_1_, is transferred to the electron (labeled as ‘2’ in Figure 1a). Another indirect transition associated with the transfer of momentum Δ*k*_2_ is unlikely to be done. Further thermalization of electrons to the bottom of the CB leads to optical heating (the shaded area on the right panel in Figure 1a). Obviously, direct/indirect absorption mechanisms are convoluted and a tunable sub-band laser probe is needed to distinguish them. Direct absorption is the dominant mechanism when resonant laser pumping, whereas indirect absorption is governed by both energy and momentum matching. Because the first limitation is dropped for electronic light scattering, as shown in Figure 1b, all indirect optical transitions become accessible, transferring both Δ*k*_1_ and Δ*k*_2_ to the electron. More importantly, inelastic broadband emission is an inherent signature of heterogeneous systems with strong spatial dispersion. These include ceramics and high-entropy crystals [25], amorphous and porous solids [26], perovskites [27], liquid crystals [25], highly-associated liquids [28], intercellular water [29], hydrogels [30], peptides and proteins [31], to name a few. This diversity underscores the fundamental importance of spatial dispersion in getting into insights of complex systems.

The increased charge population in the CB inevitably leads to optical heating through electron-phonon interaction, as seen on the right panel of Figure 1b. As a result of indirect scattering, an amplified electrical current is generated at the junction of glass and crystal [3]. At last, the most fundamental consequence of indirect scattering is an increase in the real refractive index of heterogeneous systems that can exceed the fundamental limit caused by temporal dispersion [10]. For optically transparent (*κ* ≈ 0) semiconductors the spatially varying refractive index reads as

(2) $$n^{2}(r)=1+\frac{e^{2}}{\pi^{2}m_{e}}\sum_{cv}\int_{BZ}\frac{f_{cv}^{\varkappa }\left(k\right)}{{\text{Ω}}_{cv}^{2}\left(k\right)-\omega^{2}}dk$$

where Ω_*cv*_ is a vibronic frequency corresponding to optical transitions between the Bloch electronic states $\left|v\right\rangle$ and $\left|c\right\rangle$ (Ω_*cv*_ = 0 for metals), *m*_*e*_ is the effective mass of an electron, ***ϰ*** is a light polarization direction. The oscillator strength $f_{cv}^{\varkappa }\left(k\right)$ is modified as follows

(3) $$f_{cv}^{\varkappa }\left(k\right)=\frac{2m_{e}}{\hbar }{\left|d_{cv}^{\varkappa }\left(k\right)\right|}^{2}.$$

Here *ℏ* is the Planck’s constant, $d_{cv}^{\varkappa }\left(k\right)=\left\langle c|e^{k\left(r\right)\varkappa r}\partial /\partial r|v\right\rangle$ is the transient electrical dipole moment that considers spatial dispersion. In homogeneous media, a change in the refractive index is possible at resonance only (temporal dispersion) mainly due to direct absorption (Figure 1a). In Eq. (2), integration runs over the entire Brillouin zone, and, thus, indirect transitions can contribute to the real refractive index significantly.

Depending on the energy band landscape, this process can in addition lead to optical heating of a light-scattering medium through electron-phonon interaction (Figure 1b). This process is determined by the slope of the energy band *dE*/*dk* and the population of electrons governed by Fermi–Dirac statistics. Thus, the change in overall temperature without heat transfer to the environment can be estimated using the following formula:

(4) $$\delta T=\frac{1}{k_{B}}\int_{BZ}ℱ(k-k^{\prime })\frac{dE\left(k^{\prime }\right)}{dk^{\prime }}f_{FD}\left(k^{\prime }\right)dk^{\prime }.$$

Eq. (4) indicates the fact that solids with the flat energy band valley are not heated under cw sub-band pumping.

The *k*-dependent ELS intensity is determined by the photonic density of states $ℱ\left(k\right)$ and the population of electrons, driven by Fermi–Dirac statistics $f_{FD}\left(k\right)$ , namely:

(5) $$I_{\mathrm{ELS}}\left(k\right)=C\int ℱ\left(k-k^{\prime }\right)E\left(k^{\prime }\right)f_{FD}\left(k^{\prime }\right)dk^{\prime },$$

where *C* is a constant proportional the scattering cross-section, $E\left(k\right)=\hbar \omega_{0}-E\left(k\right)$ is the scattered photon energy. In the simplest case when $ℱ\left(k-k^{\prime }\right)=I_{0}\delta \left(k-k^{\prime }\right)$ (*I*_0_ is the incident intensity, $\delta \left(k-k^{\prime }\right)$ is the Dirac-delta function), one gets

(6) $$I_{\mathrm{ELS}}\left(k\right)=C\frac{E\left(k\right)}{{\text{e}}^{\frac{E\left(k\right)-E_{p}}{k_{B}T}}+1}I_{0}≅CE\left(k\right){\text{e}}^{-\frac{E\left(k\right)-E_{p}}{k_{B}T}}I_{0},$$

where *k*_*B*_ is the Boltzmann constant, *T* is a temperature. This formula serves as a good approximation provided that high spatial homogeneity $ℱ\left(k\right)∼\delta \left(k\right)$ and $E\left(k\right)-E_{p}\gg k_{B}T$ . In Eq. (6), we introduced an additional parameter *E*_*p*_ analogous to Penn energy [32]. This seminal concept specifies the energy at which the electronic density of states is maximum throughout the entire Brillouin zone [33]. In our model, this parameter $E_{p}=\hbar \omega_{0}-E\left(k_{p}\right)$ denotes the frequency shift corresponding to the statistical mode of near-field photon momentum *k*_*p*_. This shift tends to zero for electrons trapped near the conduction band edge. Such transitions basically contribute to the appearance of the low-energy ELS peak. This is the reason why the central peak is dominant in spatially confined metals where indirect transitions near the Fermi level occur.

## 2 Results and discussion

### 2.1 A silicon atomic-force-microscopy tip

**Figure 2:**
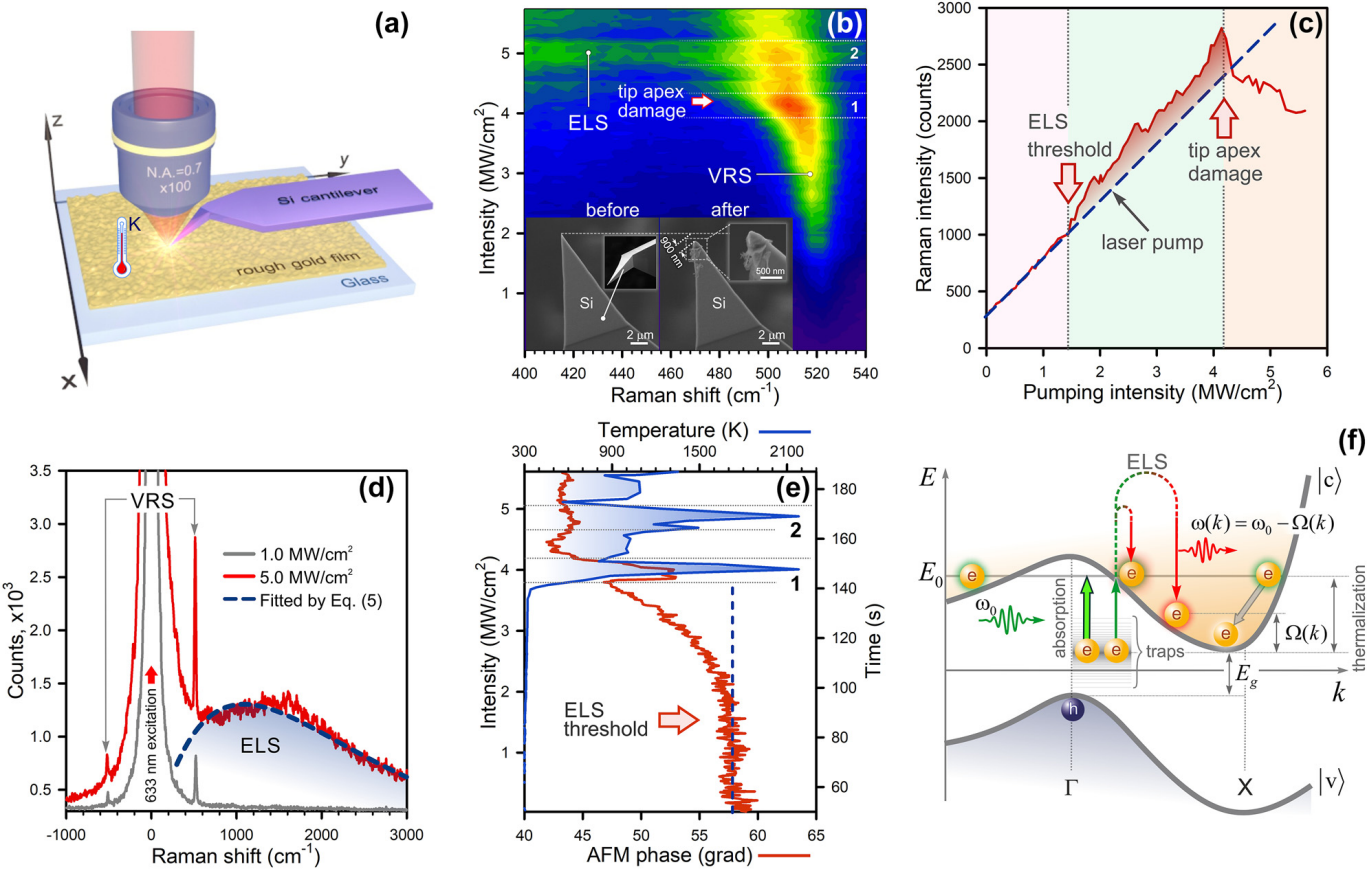
Photoheating of a silicon AFM tip apex. (a) Sketch of a TERS setup (upright configuration). (b) A Raman map of the AFM tip apex exposed to a focused laser light with different pumping intensity (the color indicates Raman intensity). The bottom inset displays SEM images of the AFM cantilever tips before and after laser impact with the intensity of 5 MW/cm^2^. (c) A plot of the VRS intensity versus the pumping intensity. (d) Raman spectra taken at the intensity of 1 MW/cm^2^ (gray) and 5 MW/cm^2^ (red) in Figure 2 (b). The dashed curve denotes a fit by Eq. (6). (e) A kinetics of the AFM cantilever phase (red) and a dependence of Raman-shift-calculated temperature versus the pumping intensity (blue). (f) The energy-momentum diagram for c-Si.

Figure 2a schematically shows a TERS setup for measuring the optical heating of spatially confined crystalline silicon (c-Si), earlier suggested in Ref. [13] In our experiment, a c-Si AFM cantilever (VIT_P, NT-MDT) top-illuminated by a focused 633 nm laser light (x100, N.A. = 0.7), oscillates in semicontact mode over a borosilicate glass substrate covered with a 50 nm Au film (TED PELLA, Inc.). We first scan the film surface with the AFM cantilever to visualize its roughness. The size of surface irregularities ranges from 1 to 2 nm (Section III, the SI). Next, the tip apex is positioned over a protrusion with the size of choice: the smaller, the stronger photon confinement is achieved [13]. Upon selecting a 1 nm high protrusion, we then record Raman spectra (exposure 1 s) and the AFM cantilever phase kinetics with a gradual increase in the pumping intensity to 5.7 MW/cm^2^ for 140 s. The optical heating is recognized by three observations: i) a temperature-dependent blueshift of the first-order vibrational Raman scattering (VRS) peak of c-Si at 521 cm^−1^ (Figure 2b), ii) high-energy ELS (Figure 2d), exhibiting a nonlinear pump-dependent intensity behavior (Figure 2c), and iii) a change in the AFM cantilever phase above the pumping intensity of 1.5 MW/cm^2^ (Figure 2e). The pump-dependent temperature of the tip apex is determined by a Raman-shift-based probe with an accuracy of 50 K (1800 grooves per mm grating) using Eq. S4 (Section IV, the SI). A calibration of the temperature probe was prior performed by temperature-dependent far-field Raman measurements in the range from 25 to 600 °C using a heating stage (Linkam Scientific Model THMS600).

Upon turning the level of 4.2 MW/cm^2^ (Figure 2b and e), the temperature steeply climbs above 2,000 K, making the tip apex melt. A close inspection of scanning electron microscopy (SEM) images of the tip apex (the inset in Figure 2b), visualized before and after laser impact, confirms its destruction within 900 nm extent. However, the AFM cantilever phase starts to respond at 1.5 MW/cm^2^ (red curve, Figure 2e). The phase shift $\Delta \varphi \approx \frac{Q}{K}\frac{\partial F}{\partial z}$ (where *K* is the spring constant, *Q* is the quality factor) is mainly driven by the normal force gradient *∂F*/*∂z* between the tip apex and the sample, that is greatly sensitive to temperature [34], [35]. It is important to note that the temperature of the entire cantilever remains constant during our experiment, except the tip apex. It follows from the fact that the resonant frequency of the AFM cantilever, equal to 300 kHz, is fixed until the tip apex is damaged, the event marked with the arrow in Figure 2b. In this figure, we highlight two regions labeled as ‘1’ and ‘2’ where giant temperature bursts are seen (Figure 2e), accompanied by ELS (Figure 2d). Thermal blinking is solely associated with tip damage. In a sense, thermal bursts in Figure 2e represent a non-stationary melting process.

This observation is in good agreement with an increase in the Raman intensity at this level (Figure 2c) that occurs due to ELS, as follows from Figure 2d. Large temperature fluctuations above 4 MW/cm^2^ (blue curve, Figure 2e) are directly related to melting and a change in the tip apex morphology. We conclude that the ELS mechanism holds promise for probing a local temperature at the hot spot.

Anomalous optical heating of spatially confined silicon is provided by near-field photons mainly generated by a rough Au film (Figure 2a). Clearly, the film itself should get heated as well. Due to the high thermal conductivity of the Au film, equal to 320 W/mK, the film is rapidly thermalized and, therefore, it is not heated.

In c-Si, that is an indirect bandgap semiconductor, the optical absorption is driven by three-body photon-phonon-electron interaction. Alternatively, this process can be activated using a near-field photon carrying not only energy, but also momentum sufficient for indirect transitions [13]. Since the electronic density of states is greatest near the band edge [14], absorption-based indirect transitions are limited because of energy band *k*-dispersion, shown in Figure 2f. In general, one should consider optical transitions to those electronic states that correspond to energy *E*_0_. Despite the fact that indirect transitions supported with large momenta are allowed, their contributions to absorption are negligible. In contrast, light scattering allows indirect transitions to all accessible states below energy *E*_0_ near the conduction band edge. This leads to broadband inelastic light scattering with a frequency shift: $\omega \left(k\right)=\omega_{0}-\Omega \left(k\right)$ (*ω*_0_ is the pumping frequency, $\Omega \left(k\right)$ is a vibronic frequency associated with the energy band), directly observed in Figure 2b and d. Eq. (6) can be used for fitting the high-energy ELS peak at 5 MW/cm^2^ (Figure 2d), by using the regularized least squares method that yields the Penn energy *E*_*p*_ = 136 meV (1,100 cm^−1^). The net temperature rise of the tip apex is mainly determined by thermal conductivity of the tip shaft served as a heatsink, whereas heat exchange with the surroundings through its surface (Kapitza resistance [36]) is negligible. It is important to notice that the mechanism of inhomogeneous broadening of a central peak at $\omega \left(k\right)\approx \omega_{0}$ is related to low-energy ELS from a rough gold film that exists even at modest pump (Figure 2d) [10], [21].

### 2.2 A gold shear-force-microscopy tip

**Figure 3:**
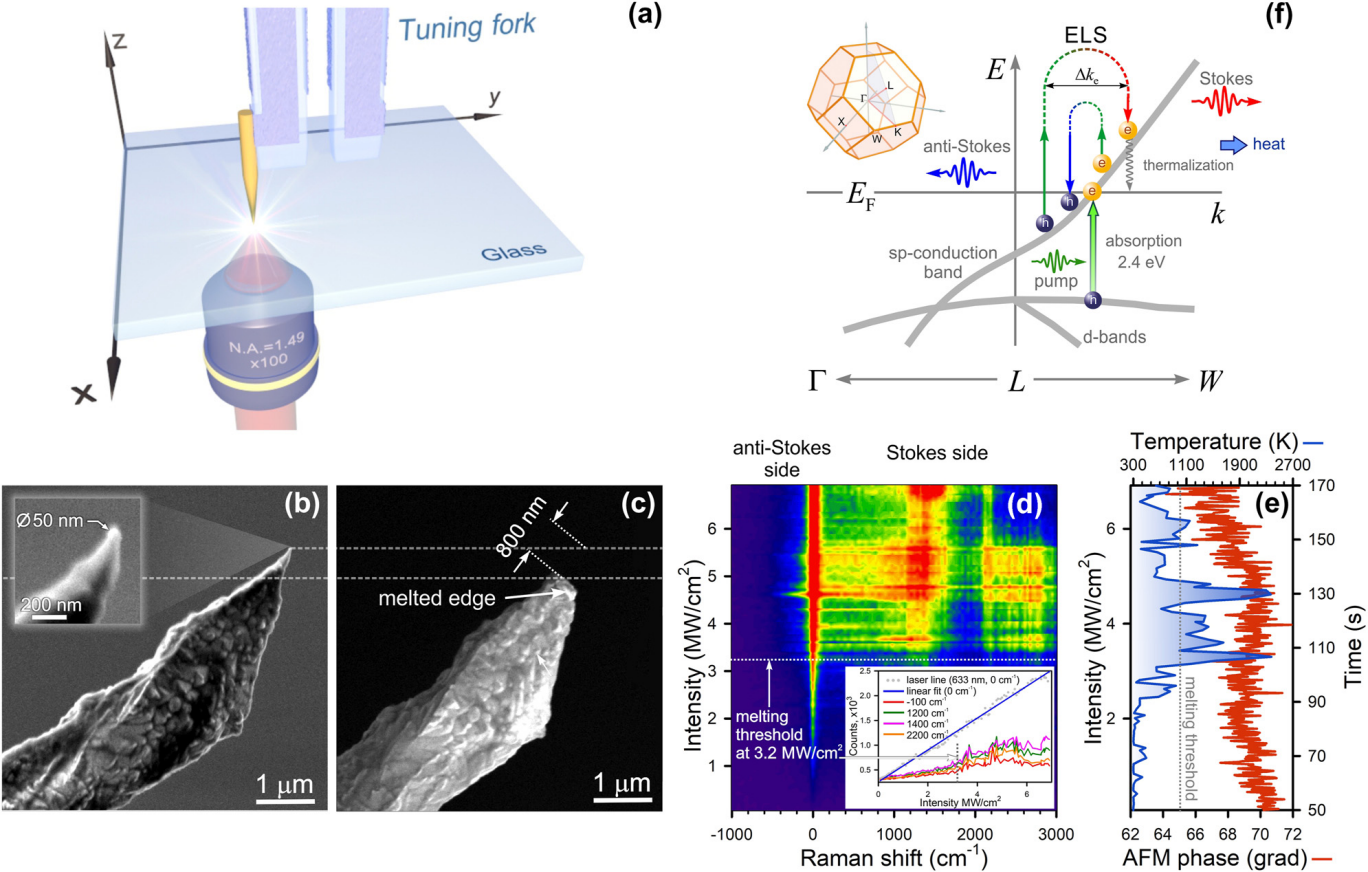
Photoheating of a gold tip apex. (a) Sketch of a TERS setup (inverted configuration). SEM images of a rough Au tips before (b) and after (c) laser illumination with the intensity of 5 MW/cm^2^. (d) A Raman map of the Au tip upon optical heating. The inset shows a dependence of Raman intensity versus pumping intensity for different wavenumbers. (e) A kinetics of the Au tip phase (red) and a dependence of temperature (blue) versus pumping intensity when illuminated by a 633 nm laser light. (f) Schematic illustration of anti-Stokes/Stokes ELS with varying electron momentum Δ*k*_e_ and interband absorption in gold. The up-left inset shows the Brillouin zone for gold bulk.

Let us now consider the spatial confinement effect for metals. Figure 3a shows a bottom-illumination TERS setup in which a rough gold tip glued to a quartz tuning fork oscillates in a plane parallel to the surface of bare coverslip with a frequency of 32 kHz. The inset in Figure 3b displays the gold tip apex of 25 nm in curvature radius. Using the same protocol as for c-Si, we reveal optical melting of the Au tip apex within 800 nm extent. This paradoxical result is explained by the fact that the melting points of Si (1,687 K) and Au (1,337 K) differ by 30 %, whereas their thermal conductivities (150 W/mK and 320 W/mK, respectively) are roughly two-fold distinct. In addition, melting points of spatially confined media can be reduced due to the size effect. In this case, a near-field photon is directly generated at the Au tip apex that is first heated and then destroyed. This effect indicates the fundamental role of expanded near-field photon momentum, enhancing light–matter interaction significantly.

We detect not only a low-energy ELS signal often found in spatially confined metals, but also a broadband high-energy ELS band (Figure 3d). The optical melting occurs upon the intensity of 3.2 MW/cm^2^. Like Si, the ELS intensity increases nonlinearly above this threshold, as seen in the inset of Figure 3d. Since the Q-factor of the tuning fork is significantly worse than that of the AFM cantilever, the shear-force-microscopy phase is less sensitive to temperature (red curve in Figure 3e). Following Sheldon et al., we used a combination of both Bose–Einstein and Fermi–Dirac statistics as a fitting function for estimating a temperature inside the Au tip apex by using the regularized least squares method [37]:

(7) $$f_{\chi }\left(E,T\right)=\chi f_{FD}\left(E,T\right)+\left(1-\chi \right)f_{BE}\left(E,T\right),$$

where *χ* is a relative contribution of both to the distribution. The melting temperature is in good agreement with the intensity threshold value at which the ELS intensity starts to increase in Figure 3d. Multiple temperature spikes are caused by dynamical destruction of the tip apex upon cw illumination impact (blue curve, Figure 3e). In contrast to semiconductors, indirect optical transitions in metals occur near the Fermi level, contributing to both anti-Stokes and Stokes signals. The balance between indirect transitions and interband absorption supports charge density oscillations within spatially confined metals, known as localized plasmon resonance.

The temperature, calculated by using Eq. (7), was found from the anti-Stokes wing, displayed in Fig. S5 (Section V, the SI). Interband absorption at 2.4 eV and above reduces the intensity of anti-Stokes scattering and, therefore, this method should be exploited with caution as temperature estimates can be biased. It is important to note that estimating temperature uncertainty is a challenging task because it requires *a priori* information on the energy band diagram, as implied by Eq. (4). In our case, the pump-dependent temperature of the gold tip apex is determined by an anti-Stokes probe with the accuracy of ca. 150 K (600 grooves per mm). Ultimately, the optical heating is determined by thermalization of charge carries and depends on the slope of the sp-conduction band (Figure 3f).

## 3 Conclusions

In this study, we have explored the optical heating of spatially confined semiconductors and metals governed by electronic light scattering rather than absorption. The underlying mechanism is the indirect transitions supported by a change in the electron momentum. This phenomenon, often encountered in SERS/TERS experiments, is still perceived as a parasitic background emission [38], [39], [40], [41]. However, an optical nanoantenna, whether a nanoparticle, a quantum dot or a defect, presents a system with strong spatial dispersion, generating broadband inelastic emission extending over several thousands of cm^−1^ [5]. This emission, that is specifically the ELS, carries important information on the spatial structure of heterogeneous media, and does not depend on their chemical composition. The indirect ELS mechanism alters an equilibrium of the electron system, leading to an increase in the real refractive index and dc-conductivity [3]. More importantly, it can heat heterogeneous media under cw illumination owing to their energy band *k*-dispersion. This phenomenon is based on expanded near-field photon momentum that provides enhanced interaction between light and spatially confined matter.

Nonlocal photonics, that studies the interaction of light and spatially confined media, is critical for engineering white light-emitting diodes and mirrorless lasers at room-temperature [3], silicon solar cells with efficiency beyond the Shockley–Queisser limit [42], [43] and optically transparent conducting electrodes [7]. A special attention should be paid to biological systems that are highly heterogeneous. The ELS expands opportunities of nonlocal photonics for optical recognition of peptide and protein conformations [44]. Of the greatest importance is non-resonant optical heating of spatially confined media under sub-band cw pumping. In particular, the ELS is a key ingredient underlying targeted thermo-optical theranostics of neurodegenerative diseases and cancer [31].

## 4 Associated content

### 4.1 Supplementary information

Details on electronic polarization in homogeneous and heterogeneous media, expanded momentum of near-field photon, atomic force microscopy measurements, Raman thermometry, electronic light scattering spectra, methods and materials.

## Supplementary Material

Supplementary Material Details

## References

[1] Ou Z. (2024). Achieving optical transparency in live animals with absorbing molecules. *Science*.

[2] Wang G. (2024). 27.09%-efficiency silicon heterojunction back contact solar cell and going beyond. *Nat. Commun.*.

[3] Kharintsev S. S., Battalova E. I., Matchenya I. A., Nasibulin A. G., Marunchenko A. A., Pushkarev A. P. (2024). Extreme electron-photon interaction in disordered perovskites. *Adv. Sci.*.

[4] Kim N. (2024). Achieving optical refractive index of 10-plus by colloidal self-assembly. *Small*.

[5] Kharintsev S. S., Battalova E. I., Noskov A. I., Merham J., Potma E. O., Fishman D. A. (2024). Photon-momentum-enabled electronic Raman scattering in silicon glass. *ACS Nano*.

[6] Maier S. (2007). *Plasmonics: Fundamentals and Applications*.

[7] Das P. (2024). Electron confinement–induced plasmonic breakdown in metals. *Sci. Adv.*.

[8] Landau L. D., Lifshitz E. M. (1984). *Electrodynamics of Continuous Media*.

[9] Kharintsev S. S., Battalova E. I., Mkhitaryan V., Shalaev V. M. (2024). How near-field photon momentum drives unusual optical phenomena: opinion. *Opt. Mater. Express*.

[10] Shim H., Monticone F., Miller O. D. (2021). Fundamental limits to the refractive index of transparent optical materials. *Adv. Mater.*.

[11] Di Mei F. (2018). Giant broadband refraction in the visible in a ferroelectric perovskite. *Nat. Photonics*.

[12] Kurman Y. (2018). Control of semiconductor emitter frequency by increasing polariton momenta. *Nat. Photonics*.

[13] Kharintsev S. S. (2024). Photon momentum enabled light absorption in silicon. *ACS Nano*.

[14] Yamaguchi M., Nobusada K. (2016). Indirect interband transition induced by optical near fields with large wave numbers. *Phys. Rev. B*.

[15] Noda M., Iida K., Yamaguchi M., Yatsui T., Nobusada K. (2019). Direct wave-vector excitation in an indirect-band-gap semiconductor of silicon with an optical near-field. *Phys. Rev. Appl.*.

[16] Shalaev V. M., Douketis C., Haslett T., Stuckless T., Moskovits M. (1996). Two-photon electron emission from smooth and rough metal films in the threshold region. *Phys. Rev. B*.

[17] Klein M. V., Cardona M. (1983). Electronic Raman scattering. *Light Scattering in Solids I*.

[18] Gass A. N., Kapusta O. I., Klimin S. A., Mal’shukov A. G. (1989). The nature of the inelastic background in surface enhanced Raman scattering spectra of coldly-deposited silver films. The role of active sites. *Solid State Commun*..

[19] Mahajan S. (2010). Understanding the surface-enhanced Raman spectroscopy “background,”. *J. Phys. Chem. C.*.

[20] Yaffe O. (2017). Local polar fluctuations in lead halide perovskite crystals. *Phys. Rev. Lett.*.

[21] Inagaki M., Isogai T., Motobayashi K., Lin K.-Q., Ren B., Ikeda K. (2020). Electronic and vibrational surface-enhanced Raman scattering: from atomically defined Au(111) and (100) to roughened Au. *Chem. Sci.*.

[22] Aouassa M. (2017). Temperature-feedback direct laser reshaping of silicon nanostructures. *Appl. Phys. Lett.*.

[23] Kharintsev S. S., Kharitonov A. V., Chernykh E. A., Alekseev A. M., Filippov N. A., Kazarian S. G. (2022). Designing two-dimensional temperature profiles using tunable thermoplasmonics. *Nanoscale*.

[24] Novotny L., Hecht B. (2012). *Principles of Nano-Optics*.

[25] Oses C., Toher C., Curtarolo S. (2020). High-entropy ceramics. *Nat. Rev. Mater.*.

[26] Canham L. (2014). *Handbook of Porous Silicon*.

[27] Kharintsev S. S. (2023). Light-controlled multiphase structuring of perovskite crystal enabled by thermoplasmonic metasurface. *ACS Nano*.

[28] Wilmshurst J. K. (1961). Lattice-type vibrations in associated liquids and the origin of anomalous Rayleigh scattering. *Nature*.

[29] Bellissent-Funel M.-C. (2016). Water determines the structure and dynamics of proteins. *Chem. Rev.*.

[30] Guo Y., Bae J., Fang Z., Li P., Zhao F., Yu G. (2020). Hydrogels and hydrogel-derived materials for energy and water sustainability. *Chem. Rev.*.

[31] Eisele Y. S. (2015). Targeting protein aggregation for the treatment of degenerative diseases. *Nat. Rev. Drug Discovery*.

[32] Penn D. R. (1962). Wave-number-dependent dielectric function of semiconductors. *Phys. Rev.*.

[33] Khurgin J. B. (2022). Expanding the photonic palette: exploring high index materials. *ACS Photonics*.

[34] Magonov S. N., Elings V., Whangbo M.-H. (1997). Phase imaging and stiffness in tapping-mode atomic force microscopy. *Surf. Sci.*.

[35] Cleveland J. P., Anczykowski B., Schmid A. E., Elings V. B. (1998). Energy dissipation in tapping-mode atomic force microscopy. *Appl. Phys. Lett.*.

[36] Sivan Y., Chu S. (2017). Nonlinear plasmonics at high temperatures. *Nanophotonics*.

[37] Hogan N., Sheldon M. (2020). Comparing steady state photothermalization dynamics in copper and gold nanostructures. *J. Chem. Phys.*.

[38] Amoruso A. B. (2024). Uncovering low-frequency vibrations in surface-enhanced Raman of organic molecules. *Nat. Commun.*.

[39] Zhang R. (2013). Chemical mapping of a single molecule by plasmon-enhanced Raman scattering. *Nature*.

[40] Lee J., Crampton K. T., Tallarida N., Apkarian V. A. (2019). Visualizing vibrational normal modes of a single molecule with atomically confined light. *Nature*.

[41] Meng Q. (2024). Local heating and Raman thermometry in a single molecule. *Sci. Adv.*.

[42] Ghasemi M., Jia B., Wen X. (2024). Lattice battery solar cells: exceeding Shockley–Queisser limit. *EcoEnergy*.

[43] Lee Y. H. (2024). Beyond the Shockley-Queisser limit: exploring new frontiers in solar energy harvest. *Science*.

[44] Ma H. (2023). Rapidly determining the 3D structure of proteins by surface-enhanced Raman spectroscopy. *Sci. Adv.*.

