# Supplementary material for: Heat generation in spatially confined solids through electronic light scattering
Source: Nanophotonics. 2025 Jun 19;14(14):2411–8. doi: 10.1515/nanoph-2025-0118 (PMC12273542; doi:10.1515/nanoph-2025-0118)
Supplement: Supplementary file 1 — Supplementary Material Details [file j_nanoph-2025-0118_suppl_001.docx]

Heat Generation in Spatially Confined Solids Through Electronic Light Scattering: Supplementary Information

S.S. Kharintsev^1*^ and E.I. Battalova^1^

^1^Department of Optics and Nanophotonics, Institute of Physics, Kazan Federal University, Kremlevskaya str., 16a, Kazan, 420008, Russia

[*skharint@gmail.com](mailto:*skharint@gmail.com)

**Section I. Electronic polarization in homogeneous and heterogeneous media**

Fig. S1 schematically shows the propagation of an incoming electromagnetic (EM) wave through a homogeneous (uniform chain) (a) and heterogeneous (non-uniform chain) (b) medium consisting of nonpolar molecules. The electronic polarization of the homogeneous medium within the wavelength equals to zero due to the coherent response of molecular polarization under the applied electric field (vertical arrows). This means that when the EM wave propagates through the homogeneous medium, the optical near-field does not arise. In an


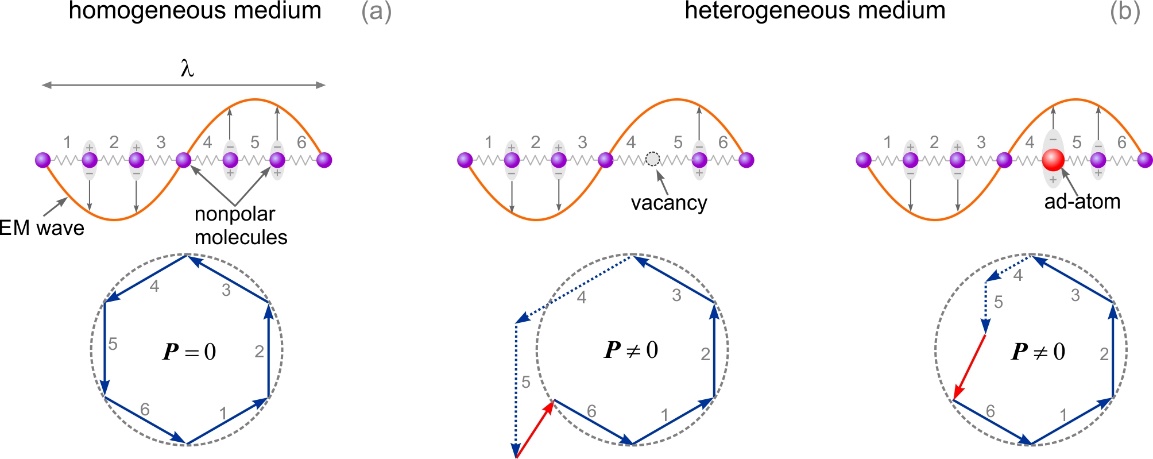


**Figure S1.** The passage of an electromagnetic (EM) wave with the wavelength $\lambda$ through homogeneous (a) and heterogeneous media (b).

opaque homogeneous medium, the attenuation of light intensity obeys the Beer-Lambert law and absorption is a dominant mechanism. In heterogeneous media enriched with diverse defects such as vacancies, ad-atoms, interstitials, etc. the electronic polarization and, therefore, the optical near-field occur. These irregularities generate near-field photons with expanded momenta due to their spatial localization at the atomic scale. Thus, indirect optical transitions contribute to the enhancement of absorption and scattering of light near defects. Indirect light scattering becomes a leading mechanism due to the non-uniform electronic density of states in the conduction band.

**Section II. The expanded momentum of near-field photon**

Fig. S2 shows the mechanism of generation of near-field photons with expanded momenta at an infinitely extended inhomogeneity (a flat interface between vacuum and a medium with the real refractive index $n$). An electromagnetic (EM) plane wave, incoming from vacuum towards the medium, meets the flat interface wherein homogeneous propagating waves in the far-field and inhomogeneous evanescent fields in the near-field are generated. Unlike the propagating EM wave, the evanescent waves cannot propagate in a homogeneous medium because they are not purely transverse and, therefore, localized within the near-field vicinity of the interface. Physically, this effect is driven by the surface polarization of charge-carriers in solids.^1^ The optical near-field photon anchored to the interface carries not only energy but also momentum.^2^ According to Heisenberg’s principle, the uncertainty of momentum is greatest at the very edge and decreases as it propagates through the homogeneous medium. The low-pass


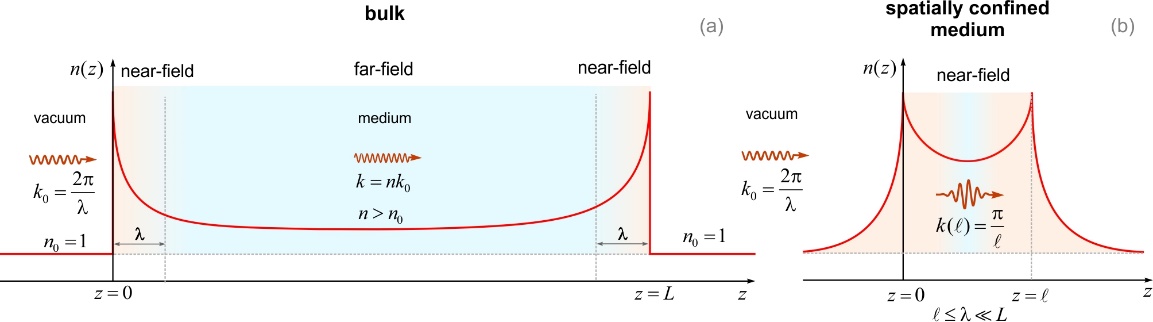


**Figure S2.** Schematic illustration of a spatial profile of the real refractive index for a bulk medium (a) and a spatially confined medium (b).

bandwidth in momentum space leads to the diffraction in the far-field.^3^ Another fundamental conclusion is the increase in the real refractive index at the interface, as schematically shown in Fig. S2 (a). In the limit of $r\gg\lambda$ a spatially varying refractive index asymptotically tends to its bulk value encountered in ray optics. It is important to emphasize that the generation of the near-field photon at the interface is followed by the expanded momentum of a propagating far-field photon in a homogeneous medium. Once the EM wave enters the medium, the near-field photon with momentum $K$ arises, which then rapidly decays over a quarter of wavelength $\lambda$ since $K=i\left| K \right|$. This is the reason why the energy conservation law $k_{0}^{2}=k^{2}+K^{2}$ requires the momentum expansion $k=nk_{0}$ ($n>1$ is the refractive index) and, therefore, $k_{0}^{2}={n^{2}k}_{0}^{2}-K^{2}$. This qualitative reasoning simplifies the conclusions, previously drawn by E. Wolf al et,^4^ in which the strength of the scattering potential $K^{2}\left( \boldsymbol{r} \right)=k_{0}^{2}\left[ n^{2}\left( \boldsymbol{r} \right)-1 \right]=-F\left( \boldsymbol{r} \right)$ is driven by a spatial profile of the real refractive index $n\left( \boldsymbol{r} \right)$ of a spatially confined object. Once the EM wave leaves the medium at $z=L$ (Fig. S2 (a)) the momentum of the far-field photon squeezes and restores its value, which it had in vacuum.

The spatial confinement of a medium within the extent $\mathcal{l}$ ($\mathcal{l\leq}\lambda<L$) (Fig. S2 (b)) radically changes light-matter interaction. In particular, the momentum of confined photon is determined by the extent of its localization rather than wavelength, $k\left( \mathcal{l} \right)=\pi/\mathcal{l}$.^2^ This means that the photon momentum can increase by several orders of magnitude, when the medium is spatially confined. This conclusion is followed by the increase in the spatially-varying real refractive index. Thus, a far-field photon propagating in a homogeneous medium solely drives direct optical transitions, whereas near-field photons generated with nanoscale constitutes of a heterogeneous medium promote indirect optical transitions due to the expanded momentum.

**Section III. Atomic force microscopy of a 50 nm Au film**

Fig. S3 a shows an AFM image of a coverslip coated with a 50 nm gold layer. The roughness of this film is visualized with a height histogram in Fig. S3 b. The statistical mode of heights is estimated to be 1.5 nm, as seen Fig. S3 b.


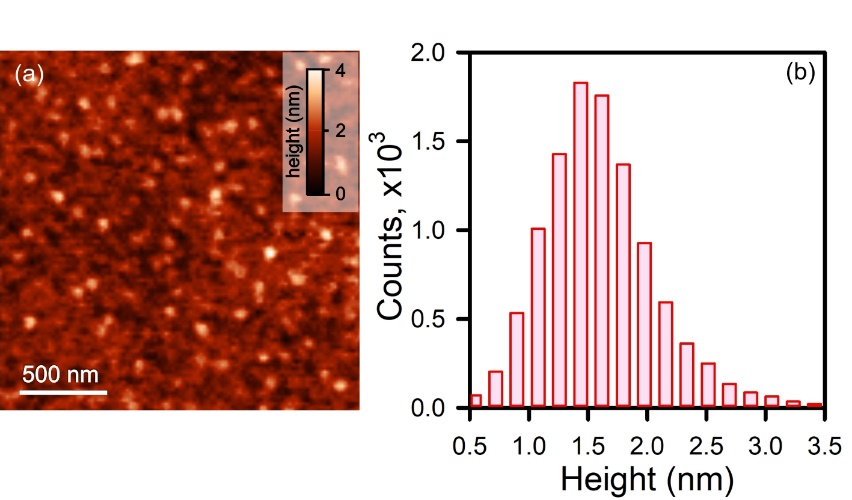


**Figure S3.** (a) AFM image of a 50 nm Au film deposited on glass, (b) The height histogram of the AFM image.

**Section IV. Raman thermometry**

Due to thermal (Boltzmann) pumping of excited vibrational levels, the lattice spacing and the lifetime of optical phonons are sensitive to temperature. This allows us to introduce intensity-, shift- and linewidth-based probes, respectively. The intensity (anti-Stokes/Stokes ratio) based probe features the most sensitive performance. However, this approach suffers from non-Boltzmann heating known as vibrational pumping.^5,6^ This effect basically impacts resonant nanostructures. In the regime of vibrational pumping, the anti-Stokes/Stokes ratio exhibits a linear dependence on the pump power and, as a result, this leads to overestimating the temperature. The linewidth-based probe is less sensitive to temperature compared to the others, but this provides more robust results for materials under stress. Among other things, the shift-based probe is more suitable for temperature estimation. For most materials, the specific Stokes peaks are blue-shifted with increasing temperature. The Stokes peak shift $\Delta(T)$ as a function of $T$ is determined as follows^7,8^

$\Delta\left( T \right)=\Omega\left( T \right)-\Omega\left( T_{0} \right)=A\left( 1+\frac{2}{e^{\frac{\hbar\omega_{0}}{2k_{B}T}}-1} \right)+B\left( 1+\frac{3}{e^{\frac{\hbar\omega_{0}}{3k_{B}T}}-1}+\frac{3}{\left( e^{\frac{\hbar\omega_{0}}{3k_{B}T}}-1 \right)^{2}} \right)$, (S4)

where $A$ and $B$ are constants specific to materials, $\omega_{0}$ is the incident photon frequency, $\Omega$ is a phonon frequency, $\hbar$ is the Plank’s constant, $k_{B}$ is the Boltzmann’s coefficient, $T$ is the


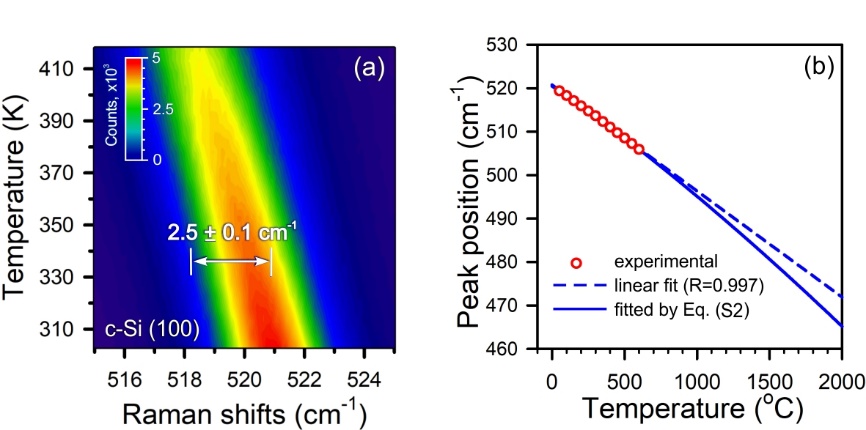


**Figure S4.** (a) Temperature-dependent Raman map of c-Si (100) with a spectral resolution of 0.1 cm^-1^ (Echelle grating), (b) a plot of the peak position vs temperature when thermal (Boltzmann) heating.

absolute temperature of a sample in unit of $K$, $T_{0}$ is equal to 0 K. For silicon, we found the following values $A=-4.391$ cm^-1^ and $B=-0.042$ cm^-1^ using temperature-dependent Raman measurements in the range from 25 to 600 ^o^C (see Fig. S4). As seen from the figure, the experimental data can be reliably fitted by a linear function within the range from 25 to 200^o^C (dashed blue curve). For larger temperatures, these should be extrapolated by using Eq. S4 (solid blue curve).

**Section V. ELS spectra of Au tip apex**


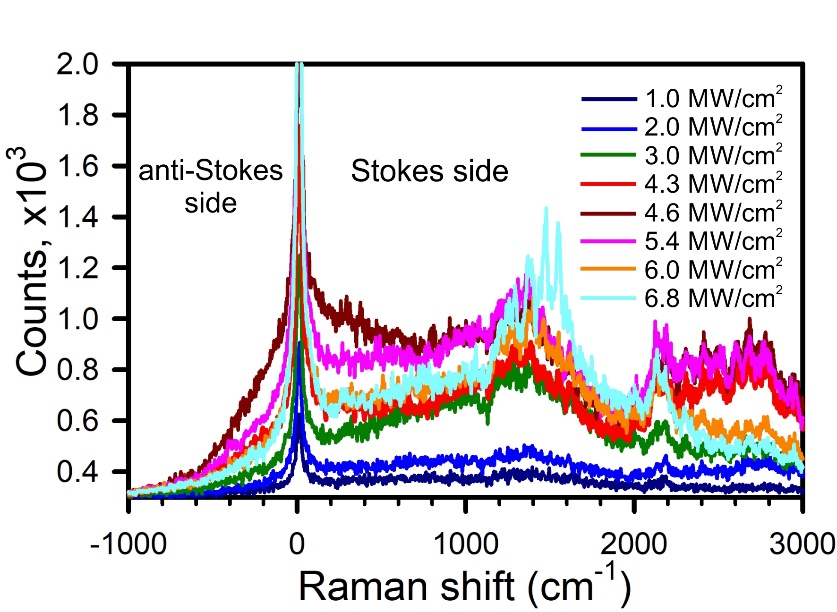


**Figure S5.** ELS spectra of a rough Au tip illuminated by a highly focused 633 nm laser light with different pumping intensity.

**Section VI. Methods and materials**

*Gold tip preparation*

Gold tips were produced with the home-built voltage controller equipped with a three-electrode electrochemical cell.^9^ This potentiostat allows one to control a current cutoff event as fast as 10 μs. A piezomanipulator immerses a 100 μm gold wire (purity: 99.99%, GoodFellow, UK) into a solution of fuming hydrochloric acid (HCl, 37%) and ethanol (C_2_H_5_OH, 96%) in a volume proportion of 1:1. A unique feature of the electrochemical cell is an inner bottom-free beaker that provides robust and stable, compared with widely used electrochemical cells, etching of a 100 *μ*m gold wire (purity: 99.99%, GoodFellow, UK) immersed. A ring gold counter-electrode with a diameter of 10 mm, which encompasses the inner beaker, is positioned by a magnitude of 10 mm in depth. Optimal etching regimes for fabricating rough tapered gold tips with the electrochemical cell are reached in the voltage range 1.5–1.7 V to be empirically found for different background electrolytes, materials of electrodes and their mutual arrangement.

*Atomic force microscopy*

The multimode scanning probe microscope Prima (NT-MDT) was utilized for visualizing a topography of light-structured silicon glasses. AFM cantilever (VIT_P) was made of antimony-doped single crystal silicon (n-type, 0.01-0.025 Ohm-cm). The tip height is 14-16 μm, the tip curvature radius is 30 nm, the resonant frequency was 300 kHz.

*Scanning electron microscopy*

The elemental composition, and morphology of the samples were studied by the Auriga Crossbeam Workstation (Carl Zeiss AG, Oberkochen, Germany), equipped with an INCA X-Max silicon drift detector (Oxford Instruments, Abingdon, UK) for energy dispersive X-ray microanalysis. For elemental analysis of Si diodes and wafers, an acceleration voltage of 5keV, an analytical working distance of 4 mm, and an electron probe current of 75 pA were used.

*Far-field and near-field Raman spectroscopy and microscopy*

Raman spectra and maps were captured with a multi-purpose analytical instrument NTEGRA SPECTRA™ (NT-MDT) in both upright and inverted configuration. The confocal spectrometer was wavelength calibrated with a crystalline silicon (100) wafer by registering the first-order Raman band at 521 cm^-1^. A sensitivity of the spectrometer was as high as ca. 2500 photon counts per 0.1 s provided that we used a 100× objective (N.A.=0.9), an exit slit (pinhole) of 100 μm and a linearly polarized light with the wavelength of 632.8 nm and the power at the sample of 10 mW. No signal amplification regimes of a Newton EMCCD camera (ANDOR) were used. Low-frequency Raman measurements were performed using a 633 nm Bragg notch filter (OptiGrate) with a spectral blocking window of 10 cm^-1^.

**References**

1. L. Novotny and B. Hecht, *Principles of Nano-Optics*, 2 ed., Cambridge: Cambridge University Press, 2012.
2. P. Bharadwaj, B. Deutsch, and L. Novotny, “Optical antennas,” *Adv. Opt. Photonics*, vol. 1, no. 3, pp. 438–483, 2009, https://doi.org/10.1364/AOP.1.000438.
3. S. S. Kharintsev, E. I. Battalova, V. Mkhitaryan, and V. M. Shalaev, “How near-field photon momentum drives unusual optical phenomena: opinion,” *Opt. Mater. Express*, vol. 14, no. 8, pp. 2017–2022, 2024, <https://doi.org/10.1364/OME.533089>.
4. E. Wolf and M. Nieto-Vesperinas, “Analyticity of the angular spectrum amplitude of scattered fields and some of its consequences,” *J. Opt. Soc. Am. A*, vol. 2, no. 6, pp. 886–890, 1985, <https://doi.org/10.1364/JOSAA.2.000886>.
5. E. C. Le Ru and P. G. Etchegoin, “Vibrational pumping and heating under SERS conditions: fact or myth?,” *Faraday Discuss*., vol. 132, pp. 63–75, 2006, <https://doi.org/10.1039/B505343A>.
6. K. Kneipp, Y. Wang, H. Kneipp, I. Itzkan, R. R. Dasari, and M. S. Feld, “Population pumping of excited vibrational states by spontaneous surface-enhanced Raman scattering,” *Phys. Rev. Lett.*, vol. 76, no. 14, pp. 2444–2447, 1996, <https://doi.org/10.1103/PhysRevLett.76.2444>.
7. G. S. Doerk, C. Carraro, and R. Maboudian, “Temperature dependence of Raman spectra for individual silicon nanowires,” *Phys. Rev. B*, vol. 80, no. 7, p. 073306, 2009, <https://doi.org/10.1103/PhysRevB.80.073306>.
8. M. Balkanski, R. F. Wallis, and E. Haro, “Anharmonic effects in light scattering due to optical phonons in silicon,” *Phys. Rev. B*, vol. 28, no. 4, 1928–1934, 1983, <https://doi.org/10.1103/PhysRevB.28.1928>.
9. S. S. Kharintsev, A. I. Noskov, G. G. Hoffmann, and J. Loos, “Near-field optical taper antennas fabricated with a highly replicable ac electrochemical etching method,” *Nanotechnology*, vol. 22, no 2, p. 025202, 2011, https://iopscience.iop.org/article/10.1088/0957-4484/22/2/025202.
